# Long non-coding RNA NEAT1/miR-338-3p axis impedes the progression of acute myeloid leukemia via regulating CREBRF

**DOI:** 10.1186/s12935-020-01182-2

**Published:** 2020-04-07

**Authors:** Song Feng, Na Liu, Xiaoguang Chen, Yufeng Liu, Jindou An

**Affiliations:** Department of Pediatrics, The First Affiliated Hospital of Zhengzhou University, No. 1 Jianshe East Road, Erqi District, Zhengzhou, 450052 Henan China

**Keywords:** NEAT1, Acute myeloid leukemia, MiR-338-3p, CREBRF

## Abstract

**Background:**

Acute myeloid leukemia (AML) is a heterogeneous hematological disease. Our purpose of the research was to investigate the regulatory influence of long non-coding RNA (lncRNA) nuclear enriched abundant transcript 1 (NEAT1)/microRNA-338-3p (miR-338-3p)/CREB3 regulatory factor (CREBRF) in AML progression.

**Methods:**

The associated RNA and protein levels were measured by quantitative real-time polymerase chain reaction (qRT-PCR) and Western blot, respectively. Cell growth was assessed through colony formation assay and 3-(4,5-dimethylthiazol-2-y1)-2, 5-diphenyl tetrazolium bromide (MTT) assay. Flow cytometry was exploited to determine the apoptosis rate. Cell migration and invasion were detected by transwell assay. The combination of miR-338-3p and NEAT1 or CREBRF was analyzed via the dual-luciferase reporter assay.

**Results:**

NEAT1 and CREBRF were down-regulated in AML tissues and cells. NEAT1 up-regulation suppressed cell growth, migration and invasion but enhanced apoptosis of AML cells. Inhibition of CREBRF reverted the NEAT1-induced effects on AML cells. Moreover, NEAT1 directly targeted miR-338-3p and miR-338-3p targeted CREBRF. NEAT1/miR-338-3p could affect cellular behaviors of AML cells via the modulation of CREBRF.

**Conclusion:**

NEAT1/miR-338-3p axis repressed the AML progression through regulating CREBRF, which might afford a favorable perspective for the AML treatment molecularly.

## Highlights

1. NEAT1 and CREBRF are down-regulated in AML tissues and cells.
2. Overexpression of NEAT1 represses cell growth, migration and invasion while promotes apoptosis of AML cells by increasing CREBRF.
3. NEAT1 targets miR-338-3p and miR-338-3p targets CREBRF.
4. NEAT1/miR-338-3p affects AML cellular processes by regulating CREBRF.

## Background

As a representative hematologic malignancy, acute myeloid leukemia (AML) is marked by the abnormal abundance of clonal myeloid progenitor cells in the bone marrow and the repression of normal hematopoiesis [1]. With the development of medical research, the modern therapies for AML have been advanced, such as chemotherapy, mutation-specific targeted therapy and hematopoietic stem cell transplantation [2–4]. Despite the appreciable survival has been obtained [5, 6], the molecular mechanism of AML still needs a thorough comprehension to develop the alternative treatment for AML.

Long noncoding RNAs (lncRNAs) are a group of the mammalian transcriptome involved in regulating various cellular behaviors of cancers at the transcriptional or post-transcriptional level [7]. Mounting studies have elaborated that lncRNAs acted as competitive endogenous RNAs (ceRNAs) to combine with miRNAs to affect gene expression in disease development. For instance, Peng et al. proclaimed that lncRNA ANCR refrained the bone formation of periodontal ligament stem cells by sponging miR-758 to up-regulate Notch2 [8]; Chen et al. purported that silencing of LINC00958 could bind to miR-330-5p to repress PAX8 in a competitive manner, thereby inhibiting the development of pancreatic cancer [9]. For AML, Wang et al. stated that lncRNA LINC00641 enhanced ZBTB20 expression via competitively binding to miR-378a to motivate cell growth and migration of AML [10]. And Peng et al. expounded that SNHG3 promoted AML cell growth by modulating the miR-758-3p/SRGN axis [11]. In addition, the down-regulation of IRAIN was found to be related to the poor prognosis of AML patients [12]. Gao et al. declared that nuclear enriched abundant transcript 1 (NEAT1), a novel lncRNA, was down-regulated in leukemia patients and cell lines, and could be used as a promising target for leukemia treatment [13]. Here, we intended to explore a specific molecular occurrence pathogenesis about AML using NEAT1 as a research object.

MicroRNAs (miRNAs) usually function as tumor regulators that reduce gene level by interacting with the 3′ untranslated region (3′UTRs) of the messenger RNA (mRNAs) of genes [14]. According to the issued reports on AML, miR-192 was testified as a tumor repressor of AML through regulating CCNT2 expression [15] while miR-183 was up-regulated in AML and contributed to cell proliferation by targeting PDCD6 [16]. Fu et al. discovered the overexpression of miR-338 in AML patients subjected to chemotherapy [17]. As a subunit of miR-338, we speculated that miR-338-3p might also expedite the progression of AML. However, its target in AML has not been found and the relation of it with NEAT1 in AML is also unknown.

CREB3 regulatory factor (CREBRF), a highly conserved protein, was reportedly acted as an anti-cancer gene of glioblastoma via obstructing the hypoxia-induced autophagy [18]. And CREBRF was shown to restrain the AML progression regulated by circRNA_0001947/miR-329-5p [19]. Nevertheless, it is unexplored whether CREBRF is a target for miR-338-3p and can be regulated by NEAT1 in AML.

Hence, this study centered on the role of NEAT1 in AML and the relational network among NEAT1, miR-338-3p and CREBRF in AML, which might help to elevate the comprehension of AML pathogenetic mechanism at the molecular level.

## Materials and methods

### Patients and tissues acquisition

In the present report, AML and normal tissues were respectively acquired from patients with AML (n = 32) and healthy donors (n = 32) at the First Affiliated Hospital of Zhengzhou University. Meanwhile, 18 tissues were collected from AML patients at the complete remission (CR) stage. The inclusion criteria for patients were shown as below: (1) Age range from 20 to 50 years, irrespective of the gender; (2) Blasts ≥ 20% of the bone marrow nucleated cells (ANC). The exclusion criteria were listed as follows: (1) Age < 18 years; (2) without complete follow-up information. Instantly, all samples were frozen in a − 80 °C ultra-low temperature freezer to provisionally conserve. This study was conducted following the signing of informed consent from all participators and the approval by the Ethics Committee of the First Affiliated Hospital of Zhengzhou University.

### Cell culture

Human bone marrow hematopoietic stem cell line CD34 and AML cell lines (KG-1, HL-60, THP-1 and U937) were respectively bought from the American Type Culture Collection (ATCC, Manassas, VA, USA) and China Center for Typical Culture Collection (Wuhan, China), then cultured in Roswell Park Memorial Institute-1640 (RPMI-1640, Gibco, Carlsbad, CA, USA) in a humidified incubator containing 5% CO_2_ at 37 °C. Specially, 10% fetal bovine serum (FBS; Gibco) and 1% penicillin–streptomycin mixture (Transgen, Beijing, China) must be supplemented into the basic medium.

### Transient transfection

KG-1 and HL-60 cells were transfected with vectors or oligonucleotides following the procedure of Lipofectamine 3000 reagent (Invitrogen, Carlsbad, CA, USA). After the sequence of NEAT1 was cloned into the pcDNA vector (NC; Invitrogen), the overexpression vector pcDNA-NEAT1 (NEAT1) was constructed. Small interfering RNA (siRNA) targeting CREBRF (si-CREBRF), siRNA negative control (si-NC), miR-338-3p mimic and inhibitor (miR-338-3p and anti-miR-338-3p), miRNA mimic and inhibitor negative control (miR-NC and anti-miR-NC) were synthesized by GenePharma (Shanghai, China).

### Quantitative real-time polymerase chain reaction (qRT-PCR)

The qRT-PCR assay was carried out as previously reported [20]. Briefly, total RNA was isolated by Trizol (Invitrogen) and reversely transcribed into cDNA via EasyScript^®^ First-Strand cDNA Synthesis SuperMix (Transgen), following the PCR reaction using TransStart^®^ Green qPCR SuperMix (Transgen) by the ABI StepOne system (Life Technologies, Carlsbad, CA, USA). Glyceraldehyde-3-phosphate dehydrogenase (GAPDH) served as the endogenous control of NEAT1 and CREBRF, as well as small nuclear RNA U6 for miR-338-3p. Primers included NEAT1 (Forward: 5′-GCCTTCTTGTGCGTTTCTCG-3′ and Reverse: 5′-TCCCAGCGTTTAGCACAACA-3′); miR-338-3p (Forward: 5′-TGCGGTCCAGCATCAGTGAT-3′ and Reverse: 5′-CCAGTGCAGGGTCCGAGGT-3′); CREBRF (Forward: 5′-GCCCTTGTTGAGCCAGATTC-3′ and Reverse: 5′-GTCTCTTTGCCACTGCACCA-3′); GAPDH (Forward: 5′-CCACTCCTCCACCTTTGAC-3′ and Reverse: 5′-ACCCTGTTGCTGTAGCCA-3′); U6 (Forward: 5′-CTCGCTTCGGCAGCACA-3′ and Reverse: 5′-AACGCTTCACGAATTTGCGT-3′). The analysis of relative expression levels was executed by the 2^−∆∆Ct^ method [21].

### Western blot

Following the obtaining of proteins by Radio-Immunoprecipitation Assay (RIPA) lysis and extraction buffer (Thermo Fisher Scientific, Waltham, MA, USA), 40 μg proteins were split on 10% sodium dodecyl sulfate–polyacrylamide gel (Invitrogen) for 2 h. 5% skim milk (Thermo Fisher Scientific) was implemented to block the non-specific binding signals after the transferring of proteins onto the polyvinylidene fluoride membranes (Thermo Fisher Scientific). Then membranes were incubated with primary antibodies: anti-CREBRF (Abcam, Cambridge, UK, ab26262, 1:1000), anti-poly-ADP-ribose polymerase (anti-PARP; Abcam, ab74290, 1:1000), anti-Cleaved PARP (Abcam, ab30264, 1:1000), anti-Cleaved caspase-3 (Abcam, ab2302, 1:1000) and anti-β-actin (Abcam, ab8227, 1:3000) for 3 h at room temperature. Subsequently, the secondary antibody (Abcam, ab205718, 1:5000) was exploited to bind to primary antibodies. 1 h later, the detection of combined signals was administered by the enhanced chemiluminescence reagent (Abcam). Ultimately, ImageLab software version 4.1 (Bio-Rad Laboratories, Hercules, CA, USA) was used for image acquisition and densitometric analysis previously [22].

### Colony formation assay

Cell resuspension was plated into the 6-well plates with 200 cells/well. About 2 weeks post-inoculation, white colonies were visibly appeared. After the fixation by methanol and staining with Giemsa (Thermo Fisher Scientific), the microscope was used for counting the colony cells.

### Cell viability detection

Cell viability was assayed every day post-transfection. 3-(4,5-dimethylthiazol-2-y1)-2,5-diphenyl tetrazolium bromide (MTT; Invitrogen) was added to cells with 20 μL per well, followed by the incubation of 200 μL dimethyl sulfoxide (DMSO; Invitrogen). Then the examination of optical density (OD) value at 490 nm was performed using a microplate reader.

### Cell apoptosis detection

Apoptosis rate was measured at 48 h post-transfection. Harvested cells were resuspended by 500 μL 1× binding buffer, then Annexin V-fluorescein isothiocyanate (Annexin V-FITC)/propidium iodide (PI) kit (BD Biosciences, San Diego, CA, USA) were exploited to dye with respective 5 μL away from light. After dying for 20 min, the apoptosis rate was analyzed via the detection of the flow cytometer (BD Biosciences).

### Detection of migration and invasion

The matrigel (Corning Life Sciences, Corning, NY, USA) was covered to the upper chamber of transwell chamber (12-wells; Corning Life Sciences) in invasion assay with no requirement in the detection of migration. Then cell suspension in serum-free medium and culture medium containing 10% FBS were severally aspirated into the upper and lower chambers. Whereafter, the number of migrated and invaded cells was calculated using the microscope following the fastening by methanol and coloring by crystal violet (Thermo Fisher Scientific).

### Dual-luciferase reporter assay

Target prediction was administrated through Starbse3.0 online software. KG-1 and HL-60 cells were transfected with pmirGLO vector (Promega, Madison, WI) recombined with wild-type (wt, with the binding sites for miR-338-3p)/mutant-type (mut, with the mutant sites for miR-338-3p) NEAT1 (NEAT1-wt and NEAT1-mut) or wt/mut 3′UTR of CREBRF (CREBRF-wt and CREBRF-mut) and miR-338-3p or miR-NC, respectively. Cells were collected 48 h later and lysed using 1 × passive lysis buffer (Promega), followed by the determination of the dual-luciferase reporter system (Promega) complying with the instruction provided by the manufacturer. The data were analyzed using renilla luciferase activity in standardization to firefly luciferase activity.

### Statistical analysis

Data were expressed as the mean ± standard deviation (SD) based on three repetitions of all assays. Data analysis and graphing relied on SPSS 19.0 and GraphPad Prism 7 softwares. Spearman’s correlation coefficient was used for analyzing the linear correlation. The comparison between two groups was conducted via Student’s *t*-test, and one-way analysis of variance followed by Tukey’s test was applied for difference analysis among multiple groups. *P* less than 0.05 was regarded as significant difference statistically.

## Results

### NEAT1 and CREBRF were down-regulated in AML tissues

**Fig. 1:**
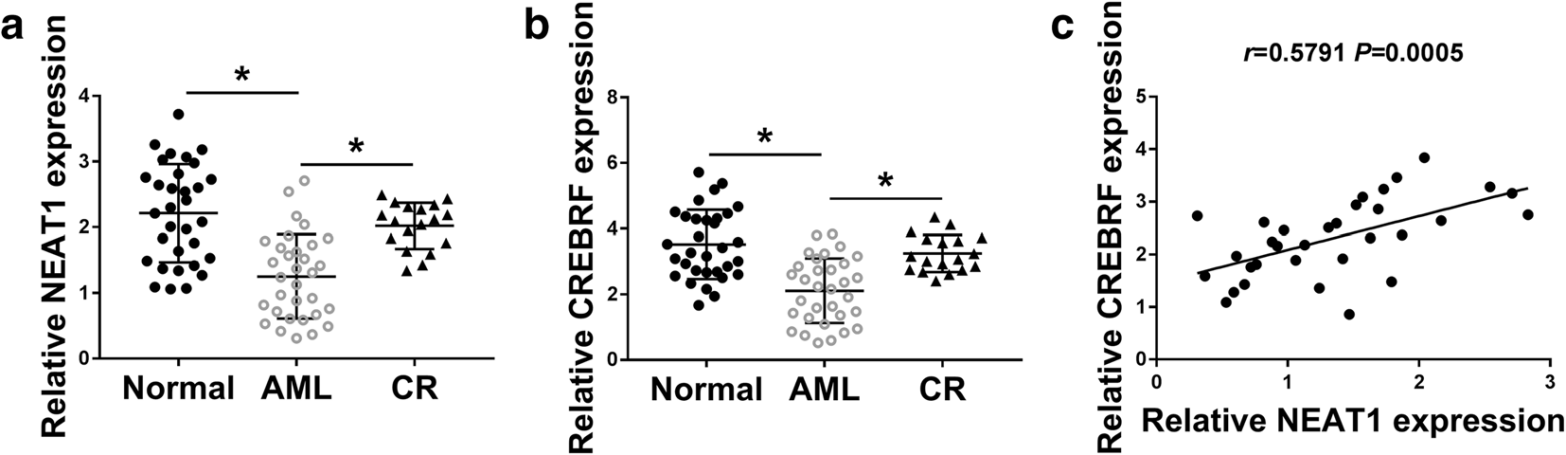
NEAT1 and CREBRF were down-regulated in AML tissues. **a**, **b** The expression of NEAT1 (**a**) and CREBRF (**b**) was assayed by qRT-PCR in normal bone marrow tissues, AML tissues and CR samples. **c** Spearman’s correlation coefficient was used for analyzing the linear relation between NEAT1 and CREBRF in AML tissues. *P < 0.05

We measured the NEAT1 and CREBRF expression in 32 pairs of normal and AML tissue samples, as well as 18 CR samples. By contrast to normal and CR tissues, the relative expression levels both of NEAT1 (Fig. 1a) and CREBRF (Fig. 1b) were remarkedly reduced in AML tissues. Meanwhile, the analysis of Spearman’s correlation coefficient showed a positive relationship (*r *= 0.5791, *P *= 0.0005) between NEAT1 and CREBRF levels in AML tissue samples (Fig. 1c). Obviously, the dysregulation of NEAT1 and CREBRF was found in AML tissues.

### NEAT1 and CREBRF levels were decreased in AML cells

**Fig. 2:**
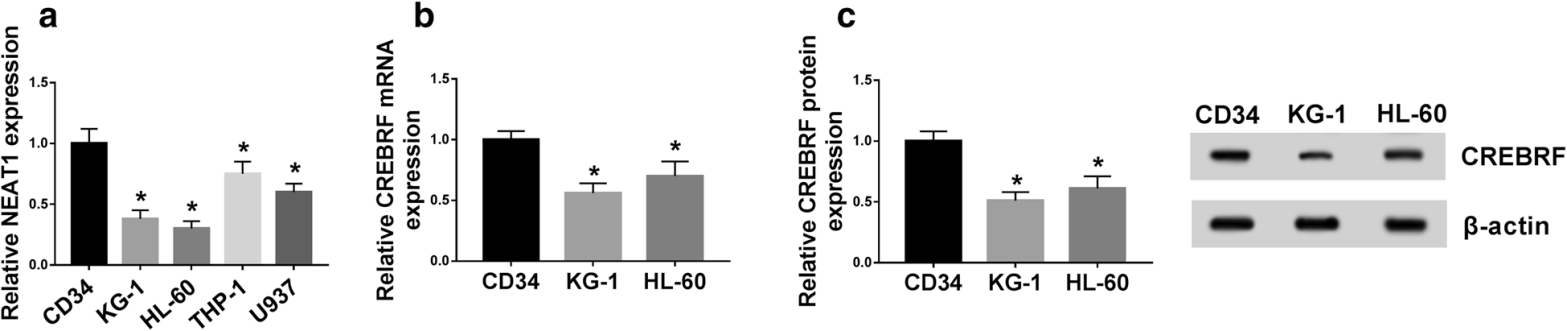
NEAT1 and CREBRF levels were decreased in AML cells. **a** QRT-PCR was applied to detect NEAT1 level in normal CD34 and AML cells. **b**, **c** The mRNA and protein levels of CREBRF in AML cells were examined by qRT-PCR and Western blot. *P < 0.05

Then we analyzed the levels of NEAT1 and CREBRF in AML cells. As described in Fig. 2a, the NEAT1 level was distinctly lower in AML cells (KG-1, HL-60, THP-1 and U937) than that in normal CD34 cells. The more conspicuous KG-1 and HL-60 cells were selected for the following experiments. The down-regulation of CREBRF mRNA (Fig. 2b) and protein (Fig. 2c) levels was also shown in KG-1 and HL-60 cells through qRT-PCR and Western blot. Therefore, the low expression of NEAT1 and CREBRF was again validated in AML cells.

### NEAT1 overexpression refrained cell growth, migration and invasion while facilitated apoptosis of AML cells

**Fig. 3:**
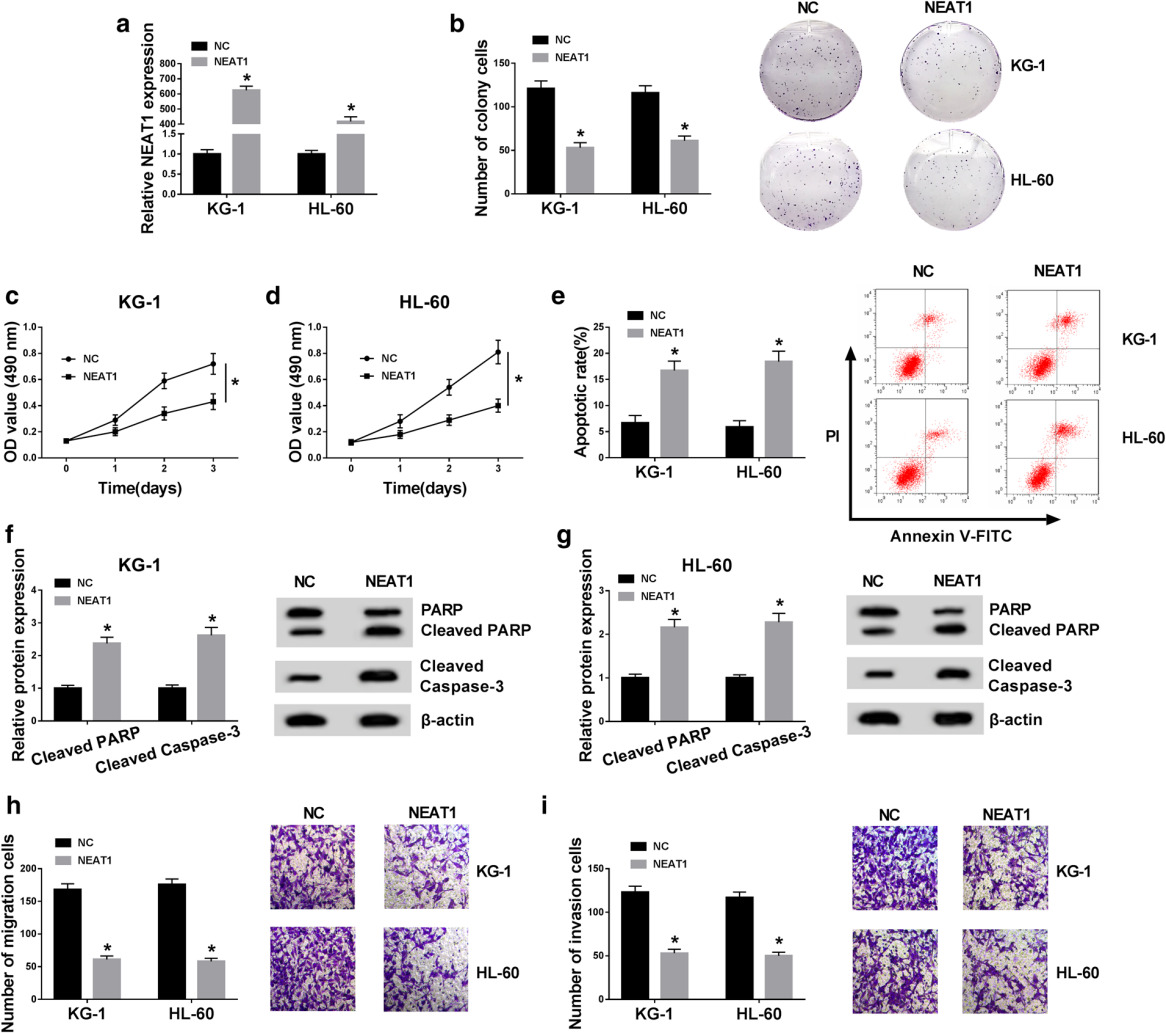
NEAT1 overexpression refrained cell growth, migration and invasion while facilitated apoptosis of AML cells. **a** The overexpression efficiency of NEAT1 was assessed through qRT-PCR. **b** Colony formation assay was administrated for the determination of colony formation in KG-1 and HL-60 cells transfected with NEAT1 or NC. **c**, **d** Cell viability was detected by MTT assay. **e** The apoptosis rate was evaluated using flow cytometry. **f**, **g** Western blot was exploited for assaying the protein levels of Cleaved PARP and Cleaved caspase-3. **h**, **i** The evaluation of cell migration and invasion abilities was conducted by transwell assay. *P < 0.05

To research the function of NEAT1 in AML, we constructed NEAT1 overexpression vector to boost the level of NEAT1 in AML cells and the transfection outcome was assessed by qRT-PCR. The NEAT1 expression in NEAT1 group was strikingly up-regulated compared to the NC group (Fig. 3a). Colony formation assay demonstrated that the colony number of KG-1 and HL-60 cells was considerably reduced after overexpression of NEAT1 (Fig. 3b). In regards to cell viability, KG-1 (Fig. 3c) and HL-60 (Fig. 3d) cells transfected with NEAT1 presented the lower OD values. After the detection of flow cytometry, we observed the up-regulation of NEAT1 triggered the elevation of apoptosis rate (Fig. 3e). PARP is one of the crucial substrates cleaved by Cleaved caspase-3 (pro-apoptosis marker) and Cleaved PARP is also an important indicator of cell apoptosis [23, 24]. Western blot suggested that the protein expression levels of both Cleaved PARP and Cleaved caspase-3 were heightened by transfection of NEAT1, indicating the promotion of apoptosis by NEAT1 up-regulation (Fig. 3f, g). In addition, the migrated (Fig. 3h) and invaded (Fig. 3i) cells was signally declined following NEAT1 overexpression. These results clarified that NEAT1 inhibited the tumorigenicity of AML in vitro.

### Down-regulation of CREBRF abrogated the NEAT1-induced effects on AML cells

**Fig. 4:**
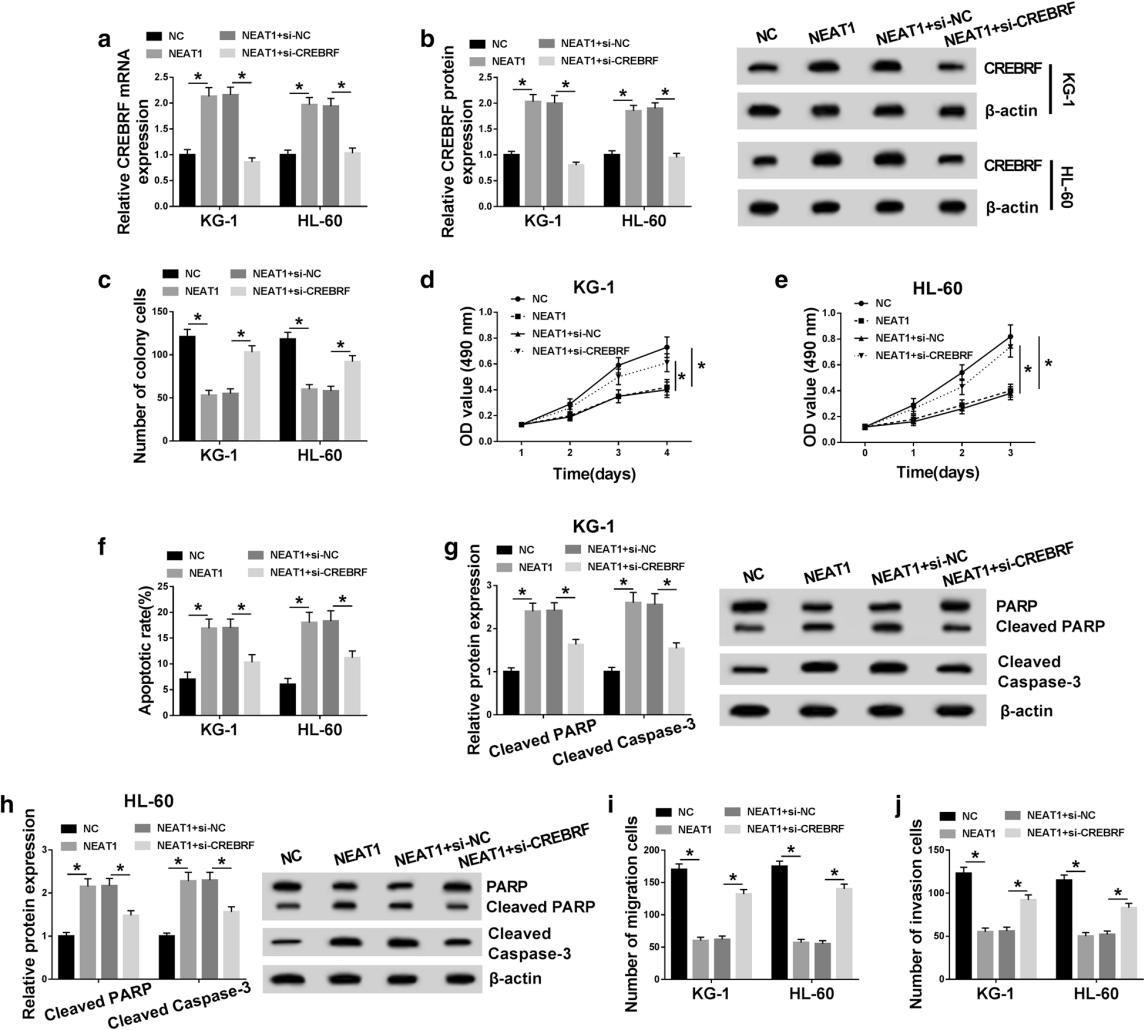
Down-regulation of CREBRF abrogated the NEAT1-induced effects on AML cells. **a**, **b** QRT-PCR and Western blot were performed to measure the mRNA and protein expression of CREBRF in KG-1 and HL-60 cells transfected with NC, NEAT1, NEAT1 + si-NC or NEAT1 + si-CREBRF. **c** The colony formation ability of transfected cells was analyzed via colony formation assay. **d**, **e** MTT was used to measure the cell viability. **f** Flow cytometry was applied to determine the apoptosis rate. **g**, **h** Cleaved PARP and Cleaved caspase-3 levels in transfected AML cells were examined by Western blot. **i**, **j** Transwell assay was implemented for evaluating cell migration and invasion abilities. *P < 0.05

CREBRF expression in KG-1 and HL-60 cells was examined by qRT-PCR and Western blot after transfection with NC, NEAT1, NEAT1 + si-NC or NEAT1 + si-CREBRF. As Fig. 4a, b displayed, CREBRF mRNA and protein levels were up-regulated in NEAT1 group, while these effects were abolished in NEAT1 + si-CREBRF group. As for cell growth, knockdown of CREBRF relieved the repressive influences on colony formation (Fig. 4c) and cell viability (Fig. 4d, e) incurred by NEAT1. Flow cytometry showed that NEAT1-induced increase of apoptosis rate was weakened by CREBRF down-regulation (Fig. 4f), which was notarized by the apoptosis-related protein detection. Western blot analysis presented that the introduction of si-CREBRF recovered the promotion of Cleaved PARP and Cleaved caspase-3 levels caused by NEAT1 (Fig. 4g, h). And co-transfection of NEAT1 and si-CREBRF had a revertible promotion of cell migration (Fig. 4i) and invasion (Fig. 4j) relative to NEAT1 transfection group. Altogether, the NEAT1-induced effects on AML cells were almost eliminated after down-regulation of CREBRF.

### NEAT1 negatively interacted with miR-338-3p and miR-338-3p directly targeted CREBRF

**Fig. 5:**
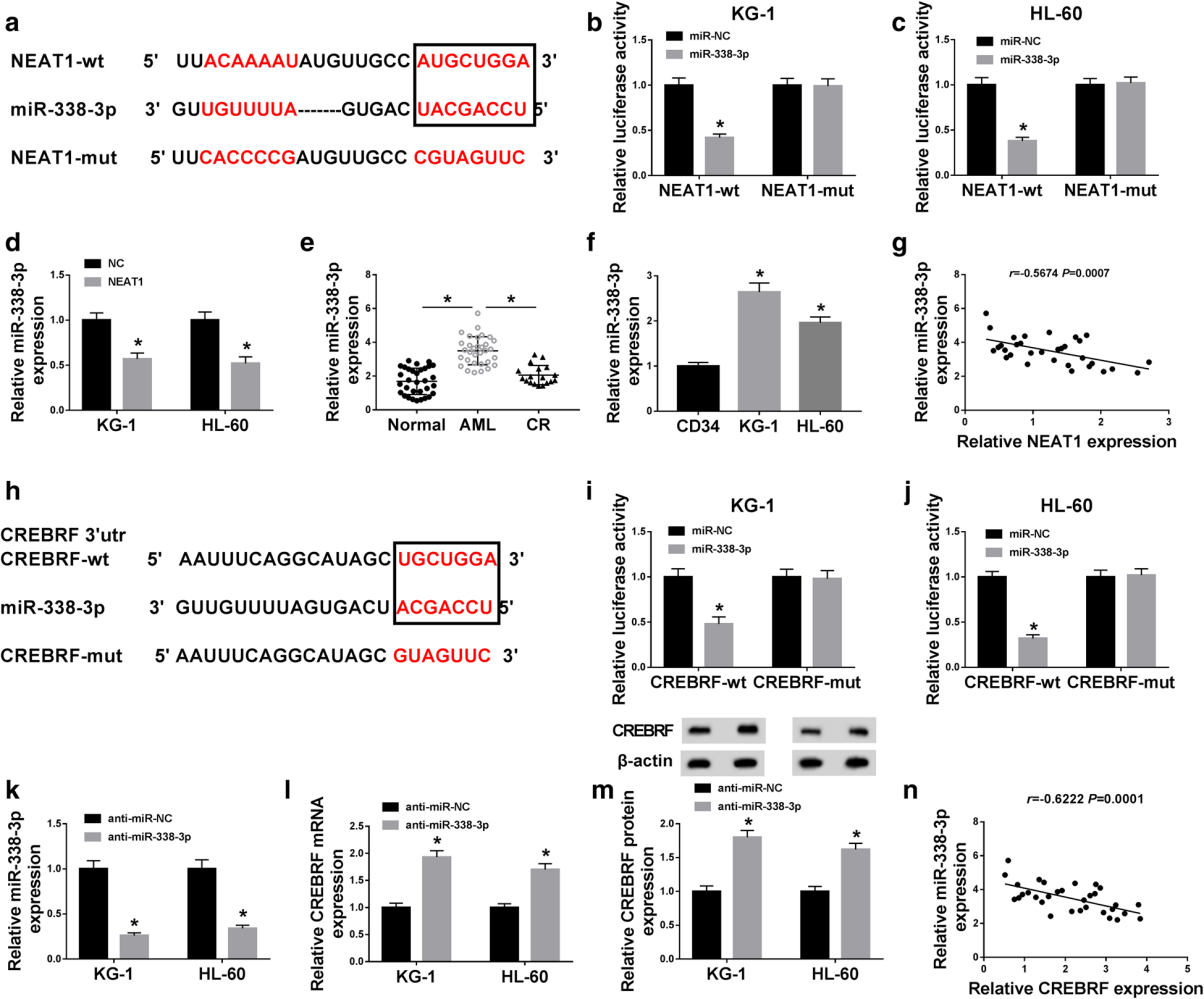
NEAT1 negatively interacted with miR-338-3p and miR-338-3p directly targeted CREBRF. **a** Starbase3.0 was carried out for the prediction of the binding sites between NEAT1 and miR-338-3p. **b**, **c** Dual-luciferase reporter assay was used for verifying the combination between NEAT1 and miR-338-3p in AML cells. **d** The miR-338-3p expression in AML cells transfected with NC or NEAT1 was detected using qRT-PCR. **e**, **f** The miR-338-3p expression in AML tissues and cells was assayed by qRT-PCR. **g** The correlation between NEAT1 and miR-338-3p in AML tissues was analyzed through Spearman’s correlation coefficient. **h** The bioinformatic analysis between miR-338-3p and CREBRF was executed by Starbase3.0 online database. **i**, **j** The affirmation of the binding between miR-338-3p and CREBRF in AML cells was affirmed by the dual-luciferase reporter assay. **k** The inhibitory efficiency of anti-miR-338-3p on miR-338-3p expression in AML cells was examined by qRT-PCR. **l**, **m** QRT-PCR and Western blot were applied for analyzing the effects of anti-miR-338-3p on CREBRF mRNA and protein levels. **n** The analysis of the relationship between miR-338-3p and CREBRF in AML tissues was administrated by Spearman’s correlation coefficient. *P < 0.05

By searching from online database Starbase3.0, we noticed that NEAT1 and miR-338-3p had the corresponding conjunct sites (Fig. 5a). To prove the interaction between NEAT1 and miR-338-3p, the dual-luciferase reporter assay was performed and the results revealed that the relative luciferase activity of KG-1 and HL-60 cells in NEAT1-wt + miR-338-3p group was much lower than that in NEAT1-mut + miR-338-3p group (Fig. 5b, c). And evidently, the miR-338-3p expression was reduced by NEAT1 overexpression (Fig. 5d). We determined the level of miR-338-3p in AML tissues and cells subsequently. As shown in Fig. 5e, f, miR-338-3p exhibited the arresting up-regulatory tendency in AML tissues and cells (KG-1 and HL-60), making a comparison with normal or CR tissues and CD34 cells. And a negative relation (*r *= − 0.5674, *P *= 0.0007) was viewed between NEAT1 and miR-338-3p in AML tissues (Fig. 5g). In the meantime, miR-338-3p could combine with the 3′UTR of CREBRF after the analysis of Starbase3.0 (Fig. 5h). Besides, transfection of miR-338-3p generated the refraining effect on the luciferase activity of CREBRF-wt group but not CREBRF-mut group in KG-1 and HL-60 cells (Fig. 5i, j). Then we used anti-miR-338-3p transfection to inhibit the expression of miR-338-3p in KG-1 and HL-60 cells (Fig. 5k), and miR-338-3p inhibitor brought about the up-regulation of CREBRF mRNA and protein expression in AML cells (Fig. 5l, m). It is interesting that miR-338-3p was also negatively associated with CREBRF (*r *= − 0.6222, *P *= 0.0001) in AML tissues (Fig. 5n). These findings validated that NEAT1 targeted miR-338-3p and CREBRF was a downstream target of miR-338-3p.

### NEAT1/miR-338-3p modulated cell growth, apoptosis, migration and invasion of AML cells by affecting CREBRF

**Fig. 6:**
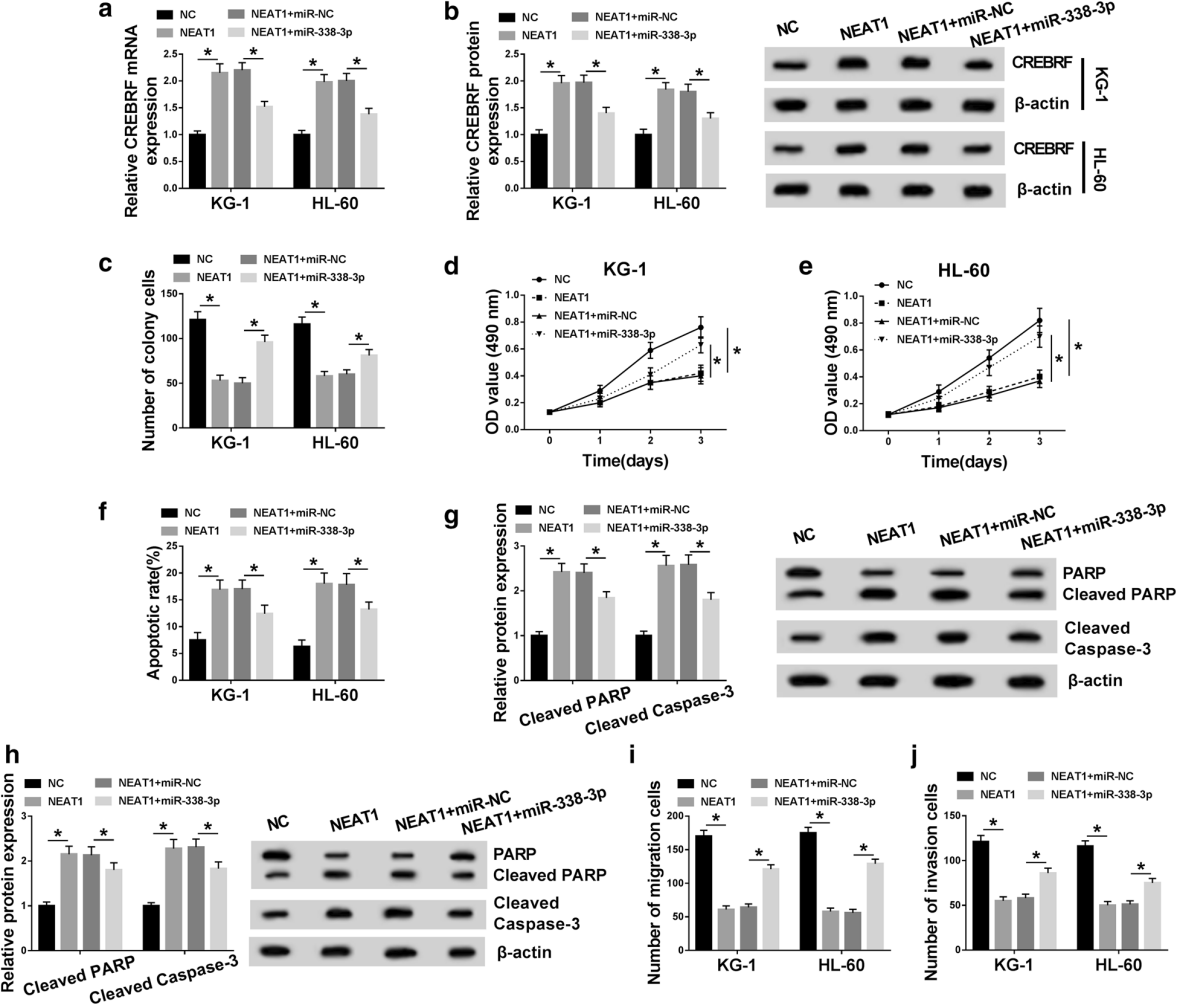
NEAT1/miR-338-3p modulated cell growth, apoptosis, migration and invasion of AML cells by affecting CREBRF. **a**, **b** QRT-PCR and Western blot were performed to measure the CREBRF mRNA and protein levels in KG-1 and HL-60 cells transfected with NC, NEAT1, NEAT1 + miR-NC or NEAT1 + miR-338-3p. **c**–**e** Cell growth was assessed using colony formation assay (**c**) and MTT assay (**d**, **e**). **f**–**h** Cell apoptosis was evaluated through apoptosis rate by flow cytometry (**f**) and the detection of Cleaved PARP and Cleaved caspase-3 protein levels by Western blot (**g**, **h**). **i**, **j** The assessment of cell migration and invasion was implemented via transwell assay. *P < 0.05

At the beginning, the correlation among NEAT1, miR-338-3p and CREBRF was researched by qRT-PCR and Western blot after transfection of NC, NEAT1, NEAT1 + miR-NC or NEAT1 + miR-338-3p. The assayed results showed that NEAT1 could facilitate CREBRF mRNA and protein expression by the targeted repression of miR-338-3p (Fig. 6a, b). Whereafter, we investigated the effects of NEAT1/miR-338-3p on cellular behaviors of AML cells. Colony formation assay and MTT denoted that NEAT1 overexpression restrained colony formation (Fig. 6c) and cell viability (Fig. 6d, e) of KG-1 and HL-60 cells via inhibiting miR-338-3p. And miR-338-3p mimic rescued the NEAT1-induced stimulative effects on apoptosis rate (Fig. 6f) and the protein levels of Cleaved PARP and Cleaved caspase-3 (Fig. 6g, h). Similarly, cell migration (Fig. 6i) and invasion (Fig. 6j) were suppressed by NEAT1 transfection, which was achieved following the decline of miR-338-3p expression. Hence, NEAT1/miR-338-3p regulated cellular behaviors of AML cells via modulating the expression of CREBRF.

## Discussion

As reported, numerous molecules have vital clinical meaning in the risk prediction, diagnosis and prognosis of AML [25]. Herein, we clarified that lncRNA NEAT1 directly interacted with miR-338-3p to enhance CREBRF expression, exerting the repression on cell growth, migration and invasion as well as the promotion of cell apoptosis in AML, indicating that NEAT1/miR-338-3p axis retarded the development of AML via up-regulating CREBRF.

Generally, lncRNAs are considered as oncogenic factors in various types of cancers. For instance, He et al. alleged that lncRNA XLOC_006390 promoted pancreatic carcinogenesis by heightening the stability of c-Myc protein [26], and CASC21 acted as a carcinogen in colorectal cancer through the regulation of miR-7-5p/YAP1 axis [27]. Also, NEAT1 was reported conductive to the bladder cancer progression by the miR-410/HMGB1 axis [28]. However, lncRNAs can also play tumorigenic roles. GAS5 inhibited osteosarcoma cell growth and metastasis by targeting miR-203a [29]. And MRPL39 hindered the progression of gastric cancer via the direct interaction with miR-130 [30]. NEAT1 was also shown to play an inhibitory action in the evolvement of several diseases. Xu et al. proposed that NEAT1 could mitigate the acute-on-chronic liver failure by blocking the TRAF6-meidated inflammatory response [31], and Zhang et al. claimed that the oxidative stress-induced vascular endothelial cell injury was suppressed by NEAT1 through the activation of the miR-181d-5p/CDKN3 axis [32]. And NEAT1 impeded the progression of osteoarthritis via the regulation of miR-181a-GPD1L axis [33]. Additionally, Zeng et al. attested that NEAT1 exacerbated cell apoptosis in chronic myeloid leukemia (CML) [34]. A recent report demonstrated that NEAT1 could reduce cell proliferation and evoke cell apoptosis of AML cells [35]. Consistently, the down-regulation of NEAT1 was confirmed in this study. Overexpression of NEAT1 had the suppressive effects on cell growth, migration, invasion and the promoted effect on cell apoptosis. The anti-tumor function of NAET1 in AML was validated in vitro, suggesting that NEAT1 overexpression might be used as a therapeutic strategy for AML treatment.

Then we found the similar low expression of CREBRF in AML tissues and cells, and CREBRF could be regulated by NEAT1. CREBRF was reported to facilitate cell proliferation of gastric cancer by mediating the AKT signal pathway [36]. Wong et al. asserted that CREBRF was relevant to the regulation of high-density lipoproteins-induced angiogenesis [37]. And Han et al. announced that CREBRF worked as an inhibitory role in AML progression [19]. In accordance with this finding, CREBRF knockdown could reverse the inhibition of NEAT1 on AML progression, implying CREBRF was a repressor of AML. And the anti-tumor effect of NEAT1 on AML depended on CREBRF.

Frequently, lncRNAs function as ceRNAs to combine with miRNAs, leading to the change of downstream gene expression which can further influence the cellular biological behaviors in cancers [38–40]. During the current research, miR-338-3p served as the intermediate connection bridge between NEAT1 and CREBRF. NEAT1 could directly target miR-338-3p and CREBRF was also identified as a target gene of miR-338-3p. As expected, NEAT1 regulated CREBRF expression level by the negative interaction with miR-338-3p. Furthermore, miR-338-3p reverted the effects of NEAT1 on AML cells, suggesting that NEAT1 hindered the AML progression through elevating CREBRF expression via competitively binding to miR-338-3p.

## Conclusion

Summarily, we gave a detailed explanation that lncRNA NEAT1/miR-338-3p refrained cell growth, migration and invasion but expedited apoptosis by modulating CREBRF in AML, manifesting that the NEAT1 exerted the suppression of AML progression by miR-338-3p/CREBRF. The NEAT1/miR-338-3p/CREBRF regulatory axis might lay a foundation for understanding the tumorigenesis molecular mechanism of AML and contributed to the clinical treatment of AML hopefully.

## Data Availability

All data generated or analyzed during this study are included in this published article.

## References

[1] Thomas D, Majeti R (2017). Biology and relevance of human acute myeloid leukemia stem cells. Blood.

[2] Burnett A, Wetzler M, Lowenberg B (2011). Therapeutic advances in acute myeloid leukemia. J Clin Oncol.

[3] Chen W, Rassidakis GZ, Medeiros LJ (2006). Nucleophosmin gene mutations in acute myeloid leukemia. Arch Pathol Lab Med.

[4] Salvatore D, Labopin M, Ruggeri A, Battipaglia G, Ghavamzadeh A, Ciceri F, Blaise D, Arcese W, Socie G, Bourhis JH (2018). Outcomes of hematopoietic stem cell transplantation from unmanipulated haploidentical versus matched sibling donor in patients with acute myeloid leukemia in first complete remission with intermediate or high-risk cytogenetics: a study from the Acute Leukemia Working Party of the European Society for Blood and Marrow Transplantation. Haematologica.

[5] Zhang Y, Zhang Y, Chen Q, Tang G, Zhang W, Yang J, Wang J, Hu X (2019). Allogeneic hematopoietic stem cells transplantation improves the survival of intermediate-risk acute myeloid leukemia patients aged less than 60 years. Ann Hematol.

[6] Reedijk AMJ, Klein K, Coebergh JWW, Kremer LC, Dinmohamed AG, de Haas V, Versluijs AB, Ossenkoppele GJ, Beverloo HB, Pieters R (2019). Improved survival for children and young adolescents with acute myeloid leukemia: a Dutch study on incidence, survival and mortality. Leukemia.

[7] Vafadar A, Shabaninejad Z, Movahedpour A, Mohammadi S, Fathullahzadeh S, Mirzaei HR, Namdar A, Savardashtaki A, Mirzaei H (2019). Long non-coding RNAs as epigenetic regulators in cancer. Curr Pharm Des.

[8] Peng W, Deng W, Zhang J, Pei G, Rong Q, Zhu S (2018). Long noncoding RNA ANCR suppresses bone formation of periodontal ligament stem cells via sponging miRNA-758. Biochem Biophys Res Commun.

[9] Chen S, Chen JZ, Zhang JQ, Chen HX, Qiu FN, Yan L, Tian YF, Peng CH, Shen BY, Chen YL (2019). Silencing of long noncoding RNA LINC00958 prevents tumor initiation of pancreatic cancer by acting as a sponge of microRNA-330-5p to down-regulate PAX8. Cancer Lett.

[10] Wang J, Liu ZH, Yu LJ (2019). Long non-coding RNA LINC00641 promotes cell growth and migration through modulating miR-378a/ZBTB20 axis in acute myeloid leukemia. Eur Rev Med Pharmacol Sci..

[11] Peng L, Zhang Y, Xin H (2019). lncRNA SNHG3 facilitates acute myeloid leukemia cell growth via the regulation of miR-758-3p/SRGN axis. J Cell Biochem.

[12] Pashaiefar H, Izadifard M, Yaghmaie M, Montazeri M, Gheisari E, Ahmadvand M, Momeny M, Ghaffari SH, Kasaeian A, Alimoghaddam K (2018). Low expression of long noncoding RNA IRAIN is associated with poor prognosis in non-M3 acute myeloid leukemia patients. Genet Test Mol Biomarkers..

[13] Gao C, Zhang J, Wang Q, Ren C (2016). Overexpression of lncRNA NEAT1 mitigates multidrug resistance by inhibiting ABCG2 in leukemia. Oncol Lett..

[14] Matoulkova E, Michalova E, Vojtesek B, Hrstka R (2012). The role of the 3′ untranslated region in post-transcriptional regulation of protein expression in mammalian cells. RNA Biol.

[15] Ke S, Li RC, Lu J, Meng FK, Feng YK, Fang MH (2017). MicroRNA-192 regulates cell proliferation and cell cycle transition in acute myeloid leukemia via interaction with CCNT2. Int J Hematol.

[16] Wang X, Zuo D, Yuan Y, Yang X, Hong Z, Zhang R (2017). MicroRNA-183 promotes cell proliferation via regulating programmed cell death 6 in pediatric acute myeloid leukemia. J Cancer Res Clin Oncol.

[17] Fu L, Qi J, Gao X, Zhang N, Zhang H, Wang R, Xu L, Yao Y, Niu M, Xu K (2019). High expression of miR-338 is associated with poor prognosis in acute myeloid leukemia undergoing chemotherapy. J Cell Physiol.

[18] Xue H, Zhang J, Guo X, Wang J, Li J, Gao X, Guo X, Li T, Xu S, Zhang P (2016). CREBRF is a potent tumor suppressor of glioblastoma by blocking hypoxia-induced autophagy via the CREB3/ATG5 pathway. Int J Oncol.

[19] Han F, Zhong C, Li W, Wang R, Zhang C, Yang X, Ji C, Ma D (2019). Hsa_circ_0001947 suppresses acute myeloid leukemia progression by sponging hsa-miR-329-5p and regulating CREBRF expression. Cancer Biol.

[20] Jing H, Qu X, Liu L, Xia H (2018). A novel long noncoding RNA (lncRNA), LL22NC03-N64E9.1, promotes the proliferation of lung cancer cells and is a potential prognostic molecular biomarker for lung cancer. Med Sci Monit..

[21] Livak KJ, Schmittgen TD (2001). Analysis of relative gene expression data using real-time quantitative PCR and the 2(−Delta Delta C(T)) method. Methods.

[22] Taylor SC, Berkelman T, Yadav G, Hammond M (2013). A defined methodology for reliable quantification of Western blot data. Mol Biotechnol.

[23] Bressenot A, Marchal S, Bezdetnaya L, Garrier J, Guillemin F, Plenat F (2009). Assessment of apoptosis by immunohistochemistry to active caspase-3, active caspase-7, or cleaved PARP in monolayer cells and spheroid and subcutaneous xenografts of human carcinoma. J Histochem Cytochem.

[24] Zhou L, Wang S, Cao L, Ren X, Li Y, Shao J, Xu L (2019). Lead acetate induces apoptosis in Leydig cells by activating PPARgamma/caspase-3/PARP pathway. Int J Environ Health Res.

[25] Prada-Arismendy J, Arroyave JC, Rothlisberger S (2017). Molecular biomarkers in acute myeloid leukemia. Blood Rev.

[26] He J, Li F, Zhou Y, Hou X, Liu S, Li X, Zhang Y, Jing X, Yang L (2020). LncRNA XLOC_006390 promotes pancreatic carcinogenesis and glutamate metabolism by stabilizing c-Myc. Cancer Lett.

[27] Zheng Y, Nie P, Xu S (2019). Long noncoding RNA CASC21 exerts an oncogenic role in colorectal cancer through regulating miR-7-5p/YAP1 axis. Biomed Pharmacother..

[28] Shan G, Tang T, Xia Y, Qian HJ (2019). Long non-coding RNA NEAT1 promotes bladder progression through regulating miR-410 mediated HMGB1. Biomed Pharmacother..

[29] Wang Y, Kong D (2018). LncRNA GAS5 represses osteosarcoma cells growth and metastasis via sponging MiR-203a. Cell Physiol Biochem.

[30] Yu MJ, Zhao N, Shen H, Wang H (2018). Long noncoding RNA MRPL39 Inhibits gastric cancer proliferation and progression by directly targeting miR-130. Genet Test Mol Biomarkers..

[31] Xu Y, Cao Z, Ding Y, Li Z, Xiang X, Lai R, Sheng Z, Liu Y, Cai W, Hu R (2019). Long non-coding RNA NEAT1 alleviates acute-on-chronic liver failure through blocking TRAF6 mediated inflammatory response. Front Physiol..

[32] Zhang M, Wang X, Yao J, Qiu Z (2019). Long non-coding RNA NEAT1 inhibits oxidative stress-induced vascular endothelial cell injury by activating the miR-181d-5p/CDKN3 axis. Artif Cells Nanomed Biotechnol..

[33] Wang Z, Hao J, Chen D (2019). Long noncoding RNA nuclear enriched abundant transcript 1 (NEAT1) regulates proliferation, apoptosis, and inflammation of chondrocytes via the miR-181a/glycerol-3-phosphate dehydrogenase 1-like (GPD1L) axis. Med Sci Monit.

[34] Zeng C, Liu S, Lu S, Yu X, Lai J, Wu Y, Chen S, Wang L, Yu Z, Luo G (2018). The c-Myc-regulated lncRNA NEAT1 and paraspeckles modulate imatinib-induced apoptosis in CML cells. Mol Cancer..

[35] Zhao C, Wang S, Zhao Y, Du F, Wang W, Lv P, Qi L (2019). Long noncoding RNA NEAT1 modulates cell proliferation and apoptosis by regulating miR-23a-3p/SMC1A in acute myeloid leukemia. J Cell Physiol.

[36] Han J, Zhang L, Zhang J, Jiang Q, Tong D, Wang X, Gao X, Zhao L, Huang C (2018). CREBRF promotes the proliferation of human gastric cancer cells via the AKT signaling pathway. Cell Mol Biol.

[37] Wong NKP, Cheung H, Solly EL, Vanags LZ, Ritchie W, Nicholls SJ, Ng MKC, Bursill CA, Tan JTM (2018). Exploring the roles of CREBRF and TRIM2 in the regulation of angiogenesis by high-density lipoproteins. Int J Mol Sci..

[38] Xie CR, Wang F, Zhang S, Wang FQ, Zheng S, Li Z, Lv J, Qi HQ, Fang QL, Wang XM (2017). Long noncoding RNA HCAL facilitates the growth and metastasis of hepatocellular carcinoma by acting as a ceRNA of LAPTM4B. Mol Ther Nucleic Acids..

[39] Feng K, Liu Y, Xu LJ, Zhao LF, Jia CW, Xu MY (2018). Long noncoding RNA PVT1 enhances the viability and invasion of papillary thyroid carcinoma cells by functioning as ceRNA of microRNA-30a through mediating expression of insulin like growth factor 1 receptor. Biomed Pharmacother..

[40] Wang LX, Wan C, Dong ZB, Wang BH, Liu HY, Li Y (2019). Integrative analysis of long noncoding RNA (lncRNA), microRNA (miRNA) and mRNA expression and construction of a competing endogenous RNA (ceRNA) network in metastatic melanoma. Med Sci Monit..

